# Subversion of the B-cell compartment during parasitic, bacterial, and viral infections

**DOI:** 10.1186/s12865-015-0079-y

**Published:** 2015-03-26

**Authors:** Gwenoline Borhis, Yolande Richard

**Affiliations:** INSERM u1016, Cochin Institute, Department of Infection, Immunity and Inflammation, 27 rue du Faubourg St-Jacques, Roussy Bldg., Paris, 75014 France; CNRS, Paris, UMR8104 France; Université Paris Descartes, Sorbonne Paris Cité, Paris, 75014 France

**Keywords:** Memory B cells, MZ B-cells, BAFF, B-reg, Virus, Parasite

## Abstract

Recent studies on HIV infection have identified new human B-cell subsets with a potentially important impact on anti-viral immunity. Current work highlights the occurrence of similar B-cell alterations in other viral, bacterial, and parasitic infections, suggesting that common strategies have been developed by pathogens to counteract protective immunity. For this review, we have selected key examples of human infections for which B-cell alterations have been described, to highlight the similarities and differences in the immune responses to a variety of pathogens. We believe that further comparisons between these models will lead to critical progress in the understanding of B-cell mechanisms and will open new target avenues for therapeutic interventions.

## Review

### Introduction

To maintain the integrity of an organism constantly challenged by pathogens, the immune system is endowed with a variety of cell types. B-cells exert a key role in both the innate and adaptive branches of immunity, through the production of protective or neutralizing antibodies (Abs), and are well suited to recognize invading pathogens or vaccine antigens (Ags). Depending on the pathogen and its route of entry, different B-cell subsets, follicular or innate B-cells, develop a specific differentiation program, namely a T-dependent (TD) or T-independent (TI) response [1]. Follicular (FO) B-cells are specialized for the adaptive response, and mainly recognize proteins, whereas marginal zone (MZ) B-cells and B1 cells support the innate response to non-protein Ags. FO B-cells populate follicles in secondary lymphoid organs, express mono-specific B-cell receptor (BCR) and require cognate interactions with Ag-activated CD4^+^ helper T-cells for initiating TD responses. This response, though slow to develop, generates a unique “serological memory” that protects from further insults by similar pathogens. In contrast, MZ B-cells reside in the spleen MZ, the sub-capsular area of lymph nodes, and the sub-epithelial area of mucosa, where they monitor invading blood-borne and mucosal pathogens [2-4]. MZ B-cells and B1 cells both express poly-specific, possibly self-reactive, BCR in combination with different innate-like receptors, which deliver co-activation signals to B-cells. Once activated they rapidly differentiate into short-lived extra-follicular plasma cells (PC) with the help of various innate cell types [2,5]. Blood-borne bacteria and viruses generally express TI and TD Ags, thereby eliciting both innate and adaptive responses.

B-cells can also exert Ab-independent regulatory functions through cytokine production and/or cognate interactions with T-cells or myeloid cells in mice and humans [6-8]. Human regulatory B-cells (B-regs), displaying different phenotypic and functional features, can improve or dampen immune responses, depending on the pathological situation. During chronic infection by hepatitis B virus (HBV) or HIV-1, B-regs inhibit the virus-specific CD8^+^ T-cell responses [9,10]. B-cells can also act as regulators of early innate immunity to virus infection. Through the expression of LTα1β2, B-cells—probably innate B-cells according to their location—are mandatory for type I interferon (IFN)-mediated survival of mice infected by cytomegalovirus [11] or vesicular stomatitis virus [12]. In these mouse models, LTα1β2-expressing B-cells provide critical signals for type I IFN production to sub-capsular CD169^+^ macrophages in the draining lymph nodes, and for virus containment [12]. Pathogens known for escaping protective immunity through antigenic variation can also use B-cells as a silent reservoir, possibly favoring pathogen spread [13-15], or can alter the phenotypes and functions of B-cells. The latter is the theme of this review; here we provide examples of parasitic, bacterial, and viral infections where B-cells with unconventional phenotypes have been identified and are thought to modulate the efficiency of pathogen-specific B- and T-cell immune responses.

## Distinct B-cell subsets

### FO B-cells in the establishment of long-lived memory

**Figure 1:**
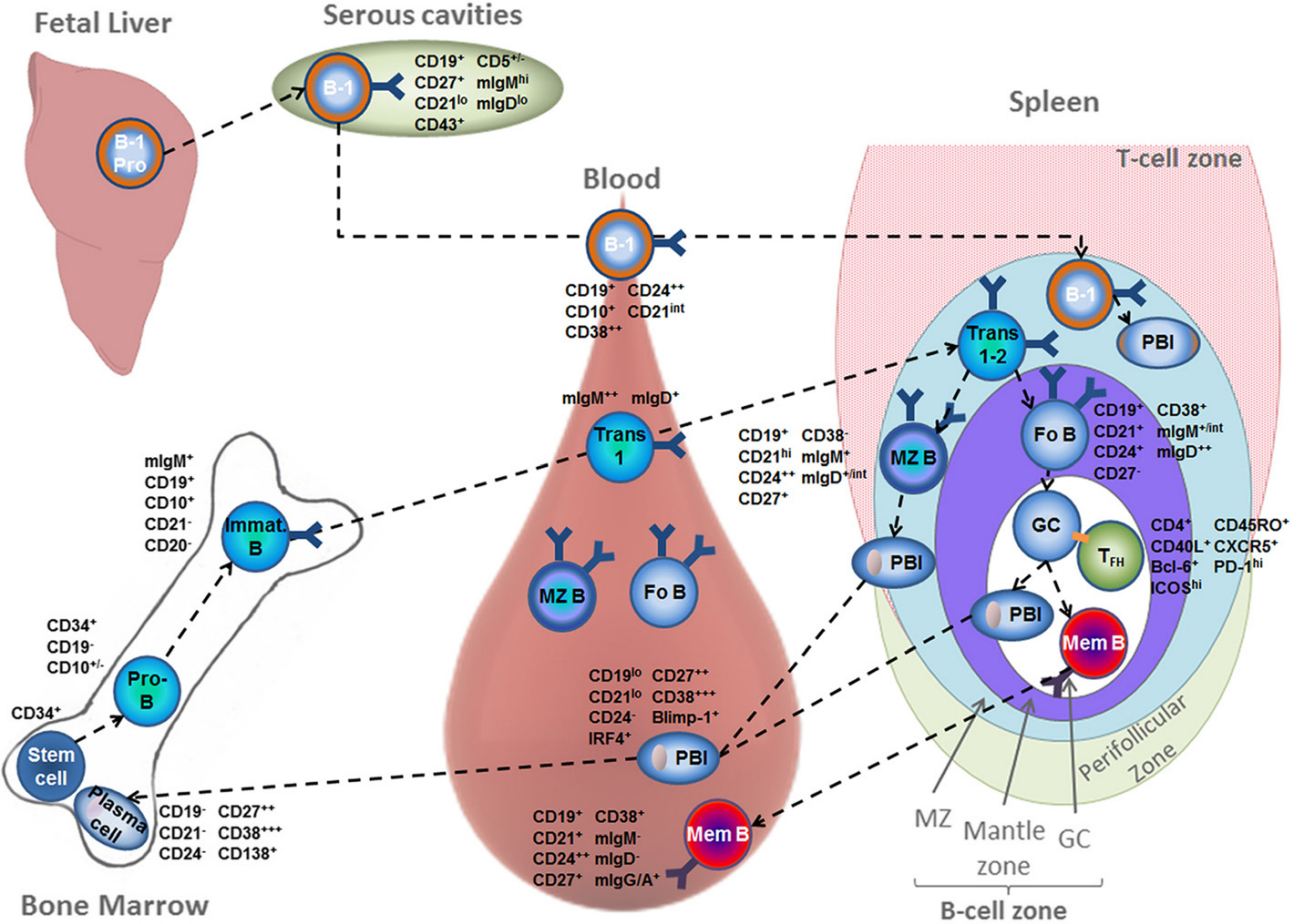
**Differentiation and trafficking of innate and follicular B-cells.** Mouse, and probably human, B-1 cells home to the serous cavities in steady state conditions and migrate to the spleen after activation by pathogens where they differentiate into natural Immunoglobulin (Ig) M-producing cells. Follicular (FO) B-cells are produced from bone marrow precursors that mature sequentially into pro-B and pre-B cells (not detailed) and immature mIgM^+^ B-cells. Immature-transitional 1 (Trans 1) B-cells migrate through the blood into spleen marginal zone (MZ) where they mature into transitional 2 (Trans 2) B-cells. Based on the balance between BCR-Notch2 signals, they next differentiate into FO or MZ B-cells. MZ B cells secrete low affinity IgM after antigenic stimulation. In the germinal center (GC), FO helper T-cells (T_FH_) support the selection and survival of B-cell clones with high-affinity BCR. Once selected, these clones differentiate into two types of effector cells, memory B-cells (Mem B) and plasma cell precursors (plasmablasts, PBl), and leave the spleen. The PBl migrate into the bone marrow and constitute a pool of long-lived plasma cells producing high-affinity Ig, whereas the Mem B migrate into extra-follicular areas in secondary lymphoid tissues.

The TD Ab response relies on the production of two kinds of effectors from FO naïve (IgD^hi^IgM^+^CD27^−^CD21^int^) B-cells: memory B-cells (MemB) and long-lived PC, who produce high-affinity Abs (Figure 1). This response occurs in lymphoid tissues, where naïve B-cells are organized in follicles in close contact with T-cell zones. Once activated by TD antigens (mainly proteins), naïve B-cells rapidly proliferate at the T/B border and generate PC that locally produce low-affinity IgM within a few days [16]. Concomitantly activated B-cells produce germinal center (GC) founder cells, which proliferate in the center of follicles. These proliferating cells no longer express BCR and become CD27^int^ and Bcl6^+^. After a set number of cell cycles, they become non-proliferating centrocytes expressing membrane switched and hyper-mutated BCR. Subsequent interactions of these centrocytes with a specialized T-cell subset, FO helper T-cells (T_FH_), and with Ag on FO dendritic cells (DC) determine the selection and survival of high-affinity B-cell clones [17-19]. Through cognate interactions and T_FH_-produced cytokines (mostly IL21, but also IL4 and IL10), selected B-cell clones differentiate into MemB and PC precursors. After their trafficking into bone marrow, PC precursors constitute a pool of long-lived PC producing high-affinity Abs whereas MemB reside in extra-follicular areas in lymphoid tissues until further encounter with similar Ags. Thus, the TD response is a fine-tuned, multistep process, which constitutes an ideal target for pathogen-induced subversion, as suggested by the altered MemB phenotypes observed during many infections, particularly chronic ones.

### Human MZ B-cells and TI Ab response

Human MZ B-cells can be distinguished from FO naïve B-cells as being SIgM^hi^CD21^hi^SIgD^+^CD23^−^CD27^+^. These memory-like B-cells express a “pre-diversified” BCR repertoire, specialized in response to various TI-1 and TI-2 Ags [4,20]. Typical TI-2 Ags are bacterial capsular polysaccharides or highly repetitive motifs found in viral capsids, which cross-link the BCR. This potent BCR activation, along with innate cell signals, elicits a rapid differentiation of MZ B-cells into extra-follicular PCs [2]. Some TI-2 Ags can transiently induce non-productive GCs that fail to generate MemB [21]. In contrast, TI-1 Ags are more heterogeneous; they include lipopeptides, liposaccharides, microbial CpG DNA, viral RNA, and some viral coat proteins. TI-1 Ags deliver a synergic activation to MZ B-cells through their BCR and toll-like receptors (TLRs), which leads to their differentiation into PC. In the absence of TLR4, human MZ B-cells can recognize various unrelated molecules from a broad spectrum of microbes through binding to their TLR2/1 and TLR2/6 complexes [22]. However, prior BCR activation is required for enhancing MZ B-cell responsiveness to TLR ligands. For example, BCR cross-linking by protein A from *Staphylococcus aureus* enhances TLR2-mediated proliferation, *in vitro* [23].

The key role of MZ B-cells in response to microbial Ags is strongly supported by studies in aged and very young individuals. Incidence of invasive pneumococcal disease increases with age, particularly in individuals that are over 65 years old [24]. Likewise, children under 2–3 years of age are highly susceptible to bacterial infections and develop minimal long-lasting protection towards polysaccharide pneumococcal vaccines [25,26]. This inefficient Ab response correlates with altered organization and functions of the spleen MZ-like area or MZ B-cells [3,27-30]. Additionally, MZ B-cells can capture and import both viral particles and high molecular weight Ags into follicles, which accelerates the initiation of the adaptive response against pathogens and widens the repertoire of Ags in the GC [31]. Thus, MZ B-cells link the innate and adaptive immune responses.

### Human B1-like cells

Numerous studies have aimed to identify human B1-like cells. Recently, Griffin *et al*. identified human B1-like cells as CD20^+^CD27^+^CD43^+^CD21^lo^CD23^−^ B-cells, with 75% being CD5^+^ [32,33]. These cells represent a small fraction of B-cells in adult blood and typically have spontaneous IgM production, skewed BCR with constitutive signaling, efficient T-cell stimulation, and an absence of CD70 or CD69 expression after stimulation [33]. Human B1-like cells are enriched in phosphorylcholine-specific B-cells [32] and produce capsular polysaccharide-specific Abs following vaccination against *Streptococcus pneumonia* (Pneumo23) [34]. Along with MZ B-cells, B1-like cells are likely involved in the TI Ab response. However, specific changes in human B1-like cells during infection have not yet been described.

## Microbial and Parasite-induced B-cell changes

Besides directly interacting with innate B-cells, microbial pathogens frequently modify their microenvironment and subvert the humoral response. Here, we discuss infections by selected widespread, mortality-causing parasites reported to induce or expand unconventional B-cell subsets (Table 1).

**Table 1:** **Summary of the main changes in B-cell subsets during parasite and viral infections**

**PATHOGENS**						
	**Plasmodium falciparum or vivax**	**Schistosomia haematobium**	**Mycobacterium tuberculosis**	**HIV-1 /SIV**	**HCV**	**HBV**
***Circulating BAFF levels***	Elevated Reduced BAFF-R expression	ND	ND	Increased during acute phase. Associated to B-cell lymphoma (chronic phase)	ND	ND
***Frequencies of B-cell subsets***						
**Immature-transtional CD10** ^**+**^ **IgM** ^**hi**^ **IgD** ^**+**^ **CD27** ^**−**^	Increased	ND	ND	Increased (chronic)	ND	ND
**MZ IgM** ^**hi**^ **IgD** ^**+**^ **CD27** ^**+**^	Reduced	Unchanged	ND	Decrease	ND	ND
**Naive IgM** ^**+**^ **IgD** ^**hi**^ **CD27** ^**−**^ **CD21int**	ND	Decreased	ND	Variable	Variable	ND
**Resting Mem IgD** ^**−**^ **CD27** ^**+**^ **CD21** ^**+**^	Decreased	Unchanged	ND	Decreased	Decreased	ND
**Activated Mem IgD** ^**−**^ **CD27** ^**+**^ **CD21** ^**−**^	ND	Increased	ND	Increased	ND	ND
**Atypical Mem SIgG/A** ^**+**^ **CD27** ^**-**^ **CD21** ^**−**^	increased FcRL4^+^, Polysaccharide IgG	Increased	ND	Increased FcRL4^+^ virus-specific Abs?	Increased, CD95^+^ IgG producers	ND
**Regulatory B-cells**	CD10^+^ Increased?	CD1d^hi^, IL10^+^, Induce T-reg Suppress IFNγ and IL17 production	CD1d^hi^CD5^+^, no IL10 or TGFβ Suppression of IL17/22 through cognate interactions	CD38^hi^CD24^hi^CD27^-^ IL10^+^, Suppress HIV-specific CD8 T-cell responses	ND	CD38^hi^CD24^hi^CD27^-^ IL10^+^, Suppress HIV-specific CD8 T-cell responses
		CD24^hi^CD27^+^ Produce TGFβ and induce Treg				
***Additionnal Remarks***	Pregenany exacerbates atypical Mem expansion	Impaired TNFα production by B-cells, even in treated patients	Impaired responses of monocytes & CD11c ^+^ DC	Impaired fonctions of DC	Impaired responses of monocytes & DC	Impaired responses of monocytes & DC
	T-cell hyporesponsiveness	Impaired responses of CD11c ^+^ DC T-cell hyporesponsiveness	T-cell hyporesponsiveness	Increased in Slan DC	Impaired CD8 T-cell responses	Impaired CD8 T-cell responses
				Strong depletion of CD4 T-cells Impaired CD8 T-cell responses		

Abs: antibodies; DC: dendritic cells; HBV: hepatitis B virus; HCV: hepatitis C virus; HIV: human immunodeficiency virus; Mem: memory; MZ: marginal zone; ND: Not documented; T-reg: regulatory T-cells; SIV: simian immunodeficiency virus.

### B-cells in human *Plasmodium* infection

Recent studies comparing various cohorts of individuals exposed to *Plasmodium* parasites, the causal agents of malaria, have revealed important changes in blood B-cell composition, in addition to T-cell hypo-responsiveness, short-lived protection by specific Abs, polyclonal B-cell activation, and an increase in total IgG during acute infection [35]. Reduced numbers of circulating MZ B-cells have been observed in children chronically exposed to *Plasmodium* parasites [36] and were associated with the well-established malaria-associated suppression of the anti-polysaccharide Ab response [37]. In adult women from high and low malaria-exposed countries, reduced proportions of blood MZ B-cells were correlated with lower levels of *Plasmodium*-specific plasma IgG [38]. Additionally, elevated plasma BAFF (B-cell activating factor belonging to tumor necrosis factor family) levels, reduced BAFF-R expression on blood B-cells, and increased numbers of circulating CD10^+^ B-cells were independently reported during controlled human malaria infection and in acutely infected children [36,39,40]. Initially considered to be B-cell precursors mobilized into blood in response to BAFF over-production, several observations suggest that these CD10^+^ B-cells might also include BAFF-induced CD10^+^ B-regs [35]: (i) in the murine model of *Babesia microti* infection, a *Plasmodium*-related model, IL10^+^ B-regs are induced that limit parasite-specific T-cell responses through expansion of regulatory T-cells (T_reg_) [41]; (ii) BAFF was shown to expand IL10^+^ B-regs in healthy mice [42]; and (iii) monocytes exposed to the soluble fraction of malaria-infected erythrocytes *in vitro* strongly express BAFF and induce B-cell proliferation and IgG secretion [43].

Increased proportions of atypical (CD21^lo^CD27^−^) MemB, which conditionally express inhibitory Fc receptor-like-4 (FcRL4), are repeatedly observed during malaria infection. In endemic areas, atypical MemB from malaria-exposed individuals express FcRL4, in combination with enhanced expression of CD19, chemokine receptors, and activation markers [44-46]. In these individuals, both classical and atypical MemB can produce neutralizing *Plasmodium*-specific IgG [45]. However, compared with classical MemB, atypical MemB are enriched in poly-reactive B-cells and recognize different *Plasmodium*-associated Ags [45]. Increased proportions of atypical MemB were also observed in women from malaria-endemic countries and correlate with the increases in *Plasmodium*-specific plasma IgG [38]. However, natural resistance to malaria in the Fulani ethnic group is correlated with increased proportions of both PC and activated MemB, thought to be the major source of protective Abs [47].

Despite similarities with tissue-like MemB [48,49], atypical MemB in malaria-exposed individuals also have features of PC precursors and might contribute to anti-malarial immunity, rather than to immune exhaustion as they do in HIV-infected patients [45,50]. Atypical MemB observed during the acute phase of controlled human malaria infection are FcRL4^−^ [40], suggesting that FcRL4 expression might be a consequence of repeated exposure to pathogen-associated Ags. Because FcRL4 reduces BCR signaling but enhances responsiveness to CpG [51], atypical MemB might be highly sensitive to *Plasmodium*-expressed non-classical TLR9 ligands [52]. Alternatively, atypical MemB might develop in response to different signaling pathways during infections by *Plasmodium* and HIV. In controlled human malaria infection, BAFF was recently proposed as a key factor in B-cell changes [40]. Similarly, BAFF overproduction was reported in macaques acutely infected with SIV [53] and in primary HIV-infected patients [54] and was associated with changes in B-cell subsets. The cellular origin of atypical MemB, the mechanisms that drive their expansion, and their capacities to release neutralizing pathogen-specific Abs during HIV infection *vs. Plasmodium* infection remain to be determined.

### B-cells in human schistosomiasis

Chronic infection with *Schistosoma haematobium* causes general immune activation, T-cell hypo-responsiveness, and impaired myeloid DC responses [55,56]. *Schistosomiasis*-infected children have increased amounts of atypical and activated MemB but decreased levels of naïve B-cells compared with uninfected children, with no differences in their resting memory or MZ B-cell frequencies. Additionally, infection by schistosomes reduces tumor necrosis factor α (TNFα) production in BCR-stimulated MemB subsets, and this might contribute to decreased pathogen-specific Th1 responses. Anti-schistosome treatment with praziquantel restores normal proportions of memory and naïve B-cells but only partially corrects TNFα production [57]. Two recent articles establish that blood CD1d^hi^CD27^−^ and CD24^hi^CD27^+^ B-regs are more numerous in schistosome-infected individuals than in healthy donors. Whereas CD1d^hi^ B-regs overexpress IL10, increase the frequency of IL10^+^ T_reg_, and suppress effector T-cell cytokines (e.g., IFNγ and IL17), the CD24^hi^ B-regs express membrane TGFβ1 and favor expansion of Foxp3^+^ T_reg_ (CD25^+^Foxp3^+^). Likely expanded through different mechanisms, these two populations synergize to dampen the schistosome-specific T-cell responses [58,59].

### B-cells and infection by *Mycobacterium tuberculosis*

Previous studies on cellular immune responses during *Mycobacterium tuberculosis* infection have established that IL17- and IL22-producing cells, CD4^+^ T-cells, and NK cells are mandatory for protective immunity against *Mycobacterium* [60-62]. However, B-cells are now considered key players in shaping the *Mycobacterium*-specific response through cognate interactions and cytokine production. They are a major component of lung granulomas in *M. tuberculosis* infection and are critical for parasite containment [63]. Human B-cells in pleural fluid and lung ectopic follicles enhance the functional activation of IL17 (Th17)- and IL22 (Th22)-expressing *M. tuberculosis*-specific T-cells but have no influence on Th1 expansion or IFNγ production [64]. A restricted CD1d^hi^CD5^+^ B-cell subset inhibits Th17/22 development through cognate interactions but not by supplying of IL10 or TGFβ. These B-cells also accumulate in the lung ectopic follicles and blood of patients with active tuberculosis. The percentage of circulating CD1d^hi^CD5^+^ B-cells within total B-cells inversely correlated with that of Th17 in these patients [65]. Although *M. tuberculosis* lysates enhance the suppressive functions of B-cells, it is not known which of the pathogen-specific Ags are responsible. In summary, different B-cell subsets with enhancing or suppressive functions modulate pathogen-specific T-cell responses and pathogen containment. Additional work is needed to identify which mechanisms (e.g., BCR, TLR2, and TLR9) control the expansion of suppressive B-cells in patients with different clinical manifestations. The putative contributions of innate (MZ or B1-like) B-cells and ectopic follicle B-cells to early and late Ab-driven protection, respectively, remain to be determined. Further study of Ab-independent B-cell functions may aid in developing new vaccine strategies.

### Multitasking B-cells during *Salmonella* infection

Bacteremia caused by *Salmonella* remains a critical human health problem, particularly in immune-compromised individuals and pregnant women. Both mouse and human B-cells are susceptible to *Salmonella* infection and can act as pathogen reservoirs, contributing to its spread [66]. The consequences of this infection on human B-cell physiology and disease progression are yet unknown. In mice, B-cells act as antigen-presenting cells required for protective T-cell responses [67]. However, more recent data show that B-regs, with PC attributes, exert immunosuppressive functions during *Salmonella* infection by supplying IL10 and/or IL35 [68]. Although the transposition of data from mice to humans is probably premature, these findings might offer interesting possibilities for treatment of *Salmonella* infections and also increase our understanding of specific B-reg expansion.

## B-cells during viral infection

Developing a vaccine against HIV-1 and understanding why the neutralizing Ab response is globally inefficient remains a challenge. Defaults in the HIV-specific Ab response were widely thought to result from a loss in CD4^+^ T-cells, but recent in-depth examinations of the B-cell population during pathogenic and non-pathogenic HIV/SIV infection have challenged this idea. These pioneering studies have largely contributed to change our global understanding of the role of B-cells.

### B-cells during HIV/SIV infection

B-cell dysfunctions are now considered to be a central feature of HIV infection and an important pathogenic mechanism [69-71]. Although B-cell hyper-activation, including centro-follicular hyperplasia, and hypergammaglobulinemia, with IgG1 being the most deregulated, were among the first symptoms described in HIV-infected patients [72-74], the role of B-cells in HIV/SIV progression has been largely underappreciated until recently. One extremely puzzling issue in HIV infection is the global inefficiency of the HIV-induced Ab response. Cumulative data reveal that circulating virus-specific Abs are detectable by one month of infection, whereas neutralizing Abs are undetectable until after 3 months. Broadly neutralizing Abs generally develop after one or two years and in only 10–30% of untreated HIV-infected patients [75]. Most neutralizing Abs are directed against HIV gp120 or gp41 proteins or their binding sites on CD4, CCR5, or CXCR4, and have features of poly-reactive or self-reactive Abs [76]. Along with the virus-specific Ab response, the humoral response to non-HIV Ags is strongly impaired, resulting in a decreased response to natural or vaccine TI and TD Ags as early as during the acute phase of infection [71,77]. Together, these data suggest that both the innate (TI) and virus-specific (TD) arms of the Ab response are impaired during HIV infection.

Chronically HIV-infected patients are reported to experience a loss in circulating MZ-like B-cells, associated with an impaired response to pneumococcal Ags [77,78]. Similarly, following infection, primary SIV-infected macaques have reduced proportions of MZ B-cells, not only in blood but also in spleen and peripheral lymph nodes [79]. Additionally, increases in circulating IgM and IgG levels and in PC numbers were observed in the spleen MZ of these animals from two weeks post-infection. Thus, virus-activated MZ B-cells likely differentiate into PC. This idea is consistent with a report showing that gp120-activated MZ-like B-cells rapidly produce IgG and IgA [80]. However, the most striking effect of HIV infection occurs within the MemB pool. Resting MemB constitute the predominant fraction of blood MemB in healthy donors, with low percentages of activated and atypical MemB [81]. In contrast, there is a paucity of resting MemB while both activated and atypical MemB are over-represented in the blood of chronically HIV-infected patients [70]. A similar decrease in resting MemB has been reported during pathogenic SIV infection [53,79,82], and this loss is concomitant with BAFF overproduction during the acute phase [53].

In chronically HIV-infected patients, atypical MemB are exhausted B-cells that express FcRL4 and other inhibitory receptors and are unresponsive to BCR triggering [50]. These cells, however, are highly responsive to TLR9 ligands and, therefore, could play a role in Ab or cytokine production. FcRL4 expression appears to protect MemB from the deleterious effects of chronic infection or inflammation [51]. Within the atypical MemB pool, HIV-specific Abs are enriched, and their production might be further enhanced by treatment with short-interfering RNA targeting FcRL4 or SIGLEC-6 [50,83]. FcRL4 expression and TGFβ1 production are induced by the binding of recombinant gp120 to the α4β7 integrin expressed by naïve B-cells [84]. Co-culture of B-cells with CD4^+^ T-cells from HIV-infected donors similarly up-regulates B-cell FcRL4 expression. Interactions between gp120 and α4β7 also reduce B-cell proliferative responses and CD80 expression [84]. The latter is consistent with our previous data showing decreased CD80, but not CD86, expression in GC B-cells from chronically HIV-infected patients [85]. Thus HIV-1 might impair both the BCR responses and co-stimulation abilities of B-cells, at least during the chronic phase of infection. Moreover, X4 gp120 proteins strongly reduce B-cell chemotaxis to not only CXCL12 but also to CCL20 and CCL21 by cross-desensitization of CCR6 and CCR7. Additionally, they induce CD62L cleavage and enhance MemB CD95 expression [86]. In summary, HIV has developed various envelope-based strategies to subvert B-cell responses, survival, and trafficking.

A key checkpoint for adaptive B-cell responses is the GC reaction leading to the generation of MemB and long-lived PC precursors. Although GC hyperplasia during pathogenic HIV/SIV infection was described long ago [85,87,88], the precise impact of the virus on GC B-cells remains elusive. We previously described the well-conserved organization and polarization of GC from the in splenic, nodular and intestinal follicles during primary SIV infection [53,79]. Levesque *et al*. observed GC fragmentation in primary HIV-infected patients [89], but generally GC involution is more frequent during the chronic and advanced phases of HIV infection when CXCR4 variants are present [85]. Similarly, early GC disruption occurs after SIV infection of Indian rhesus macaques, a model of rapid disease progression, [90] but not in the more typical models using cynomolgus or Chinese rhesus macaques [53,91].

Recent progress on the characterization of T_FH_ cells has clarified some points. First, circulating or nodular T_FH_ cells are infected by HIV/SIV similarly to, or even more strongly than other CD4^+^ T-cells, but survive longer despite continuous exposure to virus [53,92-94]. Second, during the acute phase of infection T_FH_ cells are moderately expanded in most individuals, with a correlation between tissue viral load and percentages of T_FH_ cells [95]. In contrast, chronically HIV-infected individuals and SIV-infected animals have strong inter-individual variation in their percentages of T_FH_ cells [92-94]. However, conflicting results have been reported regarding the correlation between viral load and proportions of T_FH_ during the chronic phase of infection [92,93]. Based on the proportions of CD4^+^CD45RO^+^ or CD4^+^PD1^hi^ T cells in GC, it was possible to correlate T_FH_ and GC hyperplasia in SIV-infected macaques and in the lymph nodes of chronically HIV-infected patients by *in situ* analysis [53,91,93]. In summary, during HIV/SIV infection T_FH_ cells are expanded and GCs are correctly polarized but the virus-specific response is delayed, and when it occurs, it provides relatively inefficient protection.

These paradoxical findings suggest that more subtle dysfunctions of GC B-cells, T_FH_ cells, or of their dialog occur during HIV infection and impair either the generation (within GC) or the survival and trafficking of effector B-cells (MemB or PC). The production of MemB with “alternate” phenotypes is consistent with a dysfunction of GC B-cells but might coexist with other impairments. Given that the virus is able to replicate within T_FH_ cells, gp120, Tat, and Nef proteins might be locally over-produced and interfere with the GC reaction. Indeed, Nef was shown to affect Ig class switching [96], and soluble Tat selectively increases CD40-mediated proliferation of GC B-cells [97]. In-depth phenotypic, molecular, and functional analyses of B-cell and T-cell subsets within GC and at the follicular border during the priming phase are required for a better understanding of the HIV-induced defaults that cause inappropriate Ab responses.

In this already complex situation, a new B-cell subset with regulatory functions has been recently identified. This population with a CD19^+^CD38^hi^CD24^hi^PD-L1^+^ (CD27^−^) phenotype spontaneously secretes IL10 and inhibits CD8^+^ T-cell proliferation and the HIV-specific cytotoxic response in antiretroviral-treated or untreated HIV-infected patients [9]. Besides IL10, PD-L1/PD1 interactions are assumed to critically contribute to CD8^+^ T-cell exhaustion. Patients with advanced HIV-disease also have increased proportions of circulating CD10^+^ immature-transitional B-cells [98]. Because IL7 and BAFF plasma levels were elevated in these patients [98,99], bone marrow dysfunctions and/or lymphopenia are thought to induce CD10^+^ B-cell mobilization into the periphery. Moreover, our data suggest that CD10^+^CD38^+^SIgD^+^ B-cells, which are more numerous in HIV-infected patients with a high Epstein-Barr virus (EBV) viral load and a strong depletion of resting MemB, might constitute an alternate EBV reservoir [100]. Because EBV^+^ B-cell lymphomas occur with a higher incidence in HIV-infected individuals than in the general population [101], the contribution of these CD10^+^ B-cells should be further examined.

### B-cells during hepatitis infection

Similarly to infection with HIV, infection with HBV or hepatitis C virus (HCV) is associated with polyclonal B-cell activation. When produced during the acute phase of infection, neutralizing Abs are associated with viral clearance [102]; unfortunately they frequently develop only during the chronic phase [103]. In chronically HCV-infected patients, B-cell dysfunction is reflected by IgG1 restriction, with low-titer and delayed-onset Ab responses [104]. Loss in resting MemB was associated with increased proportions of atypical MemB in HCV-infected patients, regardless of cirrhosis or hepatocellular carcinoma. This increase is likely present as early as during the acute phase of HCV infection. These atypical MemB are hypo-proliferative in response to CD40 or BCR stimulation but produce high amounts of IgG [105,106]. Increased MemB IgG production was observed in chronically HBV- and HCV-infected patients [107]. HCV is the only hepatitis infection model in which B-cell infection by particular virus quasi-species has been strongly demonstrated [108] and shown to be important for disease outcome [109]. In chronically HCV-infected patients, elevated levels of serum BAFF have been associated with autoimmunity [110]

HBV core Ag has the unique capacity to stimulate BCR in a non-Ag specific manner leading to sustained B-cell activation in chronically HBV-infected patients [107,111]. Although an extensive phenotypic and functional analysis of B-cells in HBV-infected patients is still lacking, Das *et al*. recently identified a unique subset of CD38^hi^CD24^hi^CD27^−^ B-regs, whose frequency correlates with spontaneous flares of liver disease, viral load, and serum IL10 levels. This B-cell population inhibits virus-specific CD8^+^ T-cell responses, but dampens liver inflammation through IL10 production [10].

## Conclusion

This review highlights how infections by pathogens with strongly different physiopathology lead to similar changes in B-cell phenotypes but can differently alter protective responses. As previously shown in HIV-1-infected patients, these pathogens preferentially impair the MemB compartment and frequently induce B-reg subsets that inhibit either CD4^+^ (parasites) or CD8^+^ (virus) T-cell responses. Remaining questions include the origin (MZ or FO B-cells), the mechanisms of induction, and the functional abilities of atypical MemB in various infections. Understanding the physiopathological role of B-cells during infection is important, and advances in one model of infection should benefit others.

## Abbreviations

Ab: Antibody
Ag: Antigen
BAFF: B cell–Activating Factor belonging to the TNF Family
BCR: B-Cell Receptor
B-reg: Regulatory B-cells
DC: Dendritic Cell
EBV: Epstein–Barr virus
FO: Follicular
GC: Germinal Center
HBV: Hepatitis B Virus
HCV: Hepatitis C Virus
HIV: Human Immunodeficiency Virus
Ig: Immunoglobulin
MemB: Memory B-cell
MZ: Marginal Zone
PC: Plasma Cell
SIV: Simian Immunodeficiency Virus
TD: T-cell Dependent
T_FH_: Follicular Helper T-cell
TI: T-cell independent
TLR: Toll-like Receptor
T-reg: Regulatory T-cells
